# PReS-FINAL-2137: Adalimumab monotherapy results in clinical and radiological improvement in newly diagnosed patients with juvenile spondylarthritis (JSPA)/enthesitis related arthritis(ERA)

**DOI:** 10.1186/1546-0096-11-S2-P149

**Published:** 2013-12-05

**Authors:** D Maritsi, A Soldatou, G Papaioannou, A Garoufi

**Affiliations:** 1 2nd Dept of Academic Pediatrics, "P&A Kyriakou" Childrens Hospital, Athens, Greece; 2 Department of Pediatric Radiology, "Mitera" Childrens Hospital, Athens, Greece

## Introduction

Enthesitis related arthritis (ERA) is a chronic inflammatory disorder primarily affecting the axial skeleton as well as peripheral joints and entheses. DMARD's are succesful in controling peripheral disease; however their effectiveness is diminished when axial inflammation is present. The use of anti tnfα medications in adult patients with ankylosing spondylitis is well documented. Nevertheless, data regarding their use in children with the juvenile spondylarthritis(jspa) are missing.

## Objectives

To assess the efficacy and safety of adalimumab, a fully humanized anti-tnfα monoclonal antibody, as monotherapy in jspa/ERA.

## Methods

16 patients (9 females,7 males;mean age:10 years) who met the EULAR criteria for ERA and the ASAS criteria for AS were enrolled. Following NSAID treatment failure, the patients received 24 mg/m2 of adalimumab subcutaneously every 14 days for 12 months. Patients had MRI proven sacroilitis, inflammatory lumbar pain(ILP) and were HLA-B27 positive. Outcome measures were presence of morning stiffness, duration of morning stiffness, CRP measurement, pain assessment (based on 10 cm Visual Analog Scale-VAS-) and physician assessment (based on 10 cm VAS). Mean time from diagnosis to initiation of treatment was 4 months (range 3-7 months), while from the onset of symptoms till the beginning treatment mean time was 12 months (range 5-28 months). Patients were reviewed at 3, 6, 9 and 12 months following initiation of treatment and had repeat MRI scans at 3 and 9 months.

## Results

At the beginning of the study all patients had ILP (mean pain VAS:6.8 cm), morning stiffness with a mean duration of 75 min and positive MRI findings. Mean physician VAS was 4.6 cm and mean CRP was 69 mg/dl(range: 25-102 mg/dl). At three months the mean CRP was 18 mg/dl, physician VAS was 1.9 cm, pain VAS 1.6 cm; morning stiffness improved by 80% and 57% of patients showed improved MRI findings. Reassessment at 6 months showed improvement of morning stiffness (18%) and its duration(9 min) as well as further improvement in mean physician VAS(1.2 cm) and pain VAS(1 cm). At 9 months mild further reduction in the prevalence(13%) and duration of morning stiffness(7 min) and improvement in physician VAS(0.5 cm) and pain VAS(0.7 cm) was recorded. Mean CRP was 9 mg/dl. 19% of patients showed persistent although improved MRI findings. The majority of these children had bilateral sacroilitis in the beginning of the study. At 12 months 8% had morning stiffness, and its mean duration was unchanged(7 min). Mean pain and physician VAS values were similar to those at nine months (physician VAS0.3 cm, pain VAS 0.5 cm) Mild-moderate adverse reactions were seen in 37%.

## Conclusion

This is a novel study in the pediatric population showing that adalimumab monotherapy in early stages of jspa/ERA is effective and safe. Adalimumab leads to early remission as defined by reduction of pain and stiffness. Overall, patient improvement is gradual and sustained. No further improvement was noted beyond 9 months of treatment. MRI findings show slower improvement; however 19% of patients had radiological evidence of inflammation at nine months. More studies are required to prove its long term benefit and safety.

## Disclosure of interest

None declared.

